# Manikin-based practice combined with scenario-based simulation for cardiopulmonary resuscitation training among middle school students: study protocol for a cluster randomized controlled trial

**DOI:** 10.3389/fpubh.2026.1878058

**Published:** 2026-07-08

**Authors:** Xiaofei Hao, Qiyu Li, Tingting Cui, Jing Wang, Liping Gu, Xiaoxuan Gao, Yumin Wang, Fang Gao

**Affiliations:** 1 Inner Mongolia Autonomous Region Maternal and Child Health Care Hospital, Hohhot, China; 2 School of Medical Humanities, China Medical University, Shenyang, China; 3 School of Nursing, Henan University of Science and Technology, Luoyang, China; 4 School of Public Health, Peking University, Beijing, China; 5 Stomatology Hospital, School of Stomatology, Zhejiang University School of Medicine, Hangzhou, China; 6 School of Humanities and Social Sciences, Harbin Medical University, Harbin, China

**Keywords:** cardiopulmonary resuscitation, factorial design, middle school students, randomized controlled trial, scenario-based simulation

## Abstract

**Background:**

Out-of-hospital cardiac arrest is highly fatal, and the timely delivery of high-quality cardiopulmonary resuscitation (CPR) is critical for improving survival outcomes. However, current CPR training for middle school students is still mainly based on traditional teaching methods, with limited real-life scenario simulation, making it difficult to effectively enhance skill application and emergency response capacity. Manikin-based training and scenario-based drama review, as important forms of experiential learning, may offer a promising approach to optimizing CPR training models. However, high-quality evidence regarding their effectiveness in middle school populations remains limited.

**Methods:**

A single-blind cluster randomized controlled trial with a 2 × 2 factorial design will be conducted in a middle school in Hohhot, Inner Mongolia Autonomous Region. A total of 160 eligible middle school students will be recruited and randomly assigned in a 1:1:1:1 ratio to four groups: conventional CPR training group, manikin-based training group, scenario-based drama review group, and combined manikin-based training plus scenario-based drama review group. Each training session will last 90 min. The primary outcomes are CPR-related knowledge score and skill performance score. Secondary outcomes include self-efficacy (behavioral performance and evaluation), behavioral intention, course satisfaction, and skill retention rate. Data will be collected at Baseline assessment (T0), immediate post-first training assessment (T1), 3-month post-first training assessment (T2), immediate post-second training assessment (T3), 3-month post-second training follow-up (T4).

**Discussion:**

This study applies a CPR training model integrating manikin-based practice and scenario-based drama review, and systematically evaluates the independent effects and interaction effects of different intervention components using a factorial design. The expected findings will provide more practical and effective intervention strategies for CPR training among middle school students, and offer empirical evidence for optimizing school-based first aid education systems and informing relevant policy development.

**Clinical trial registration:**

https://www.chictr.org.cn, Identifier, ChiCTR2600124162.

## 1 Introduction

### 1.1 Background

Out-of-hospital cardiac arrest (OHCA) is a major public health concern worldwide, characterized by a relatively low incidence but a high mortality rate (1). In Europe and North America, the annual incidence of OHCA ranges from 55 to 113 cases per 100,000 population. In contrast, China reports a higher incidence of OHCA but substantially poorer outcomes, with a survival rate of only 1.6%, which is markedly lower than that reported in the United States (9%) and Europe (18%) (2). Timely and high-quality early intervention is critical for improving survival among patients with cardiac arrest (3). When effective cardiopulmonary resuscitation (CPR) and defibrillation are initiated within 4 min of cardiac arrest, survival rates can reach 49–75% (4). A growing body of evidence indicates that bystander-initiated CPR significantly improves survival outcomes in patients experiencing cardiac arrest (5).

According to data from the National Emergency Medical Services Information System (NEMSIS) in the United States, more than 23,000 individuals under the age of 18 experience OHCA each year (6). It has been suggested that at least 15% of the population needs to be trained in CPR in order to achieve a meaningful improvement in OHCA survival rates (7). Schools are widely recognized as ideal settings for delivering CPR education at a population level. The World Health Organization, through its “Kids Save Lives” initiative, recommends that all schools worldwide provide at least 2 h of CPR training annually to individuals aged 11 years and older (6). Middle school students are at a developmental stage characterized by increasing social engagement and a growing sense of responsibility. They possess strong learning and imitation abilities, making them a key target population for CPR knowledge dissemination and skill training. The school environment, as a centralized educational setting, enables the large-scale and systematic delivery of first aid training. Moreover, adolescents in this age group demonstrate high receptivity to hands-on skills and relatively strong retention, which contributes to both the effectiveness and sustainability of training outcomes. In addition, middle school students are often present at the scene of emergencies within school environments and therefore have the potential to act as “first responders.” However, their current levels of first aid knowledge and practical skills remain insufficient, increasing the likelihood of missed opportunities for timely intervention. Through a “student–family–community” dissemination pathway, combined with scenario-based and experiential learning approaches, CPR training can not only enhance students’ rescue competencies and psychological readiness but also contribute to improving overall public emergency preparedness and health literacy.

At present, cardiopulmonary resuscitation (CPR) training for middle school students is still primarily delivered through traditional methods such as lectures, demonstrations, and paper-based exercises. However, substantial limitations remain in the simulation of real-life emergency scenarios (8). Given that cardiac arrest is characterized by sudden onset, rapid progression, and an extremely limited window for optimal resuscitation, even a delay of a few seconds may adversely affect patient outcomes. As a result, conducting on-site teaching using real patients presents considerable challenges in terms of both safety and feasibility. In recent years, teaching approaches that combine manikin-based practice with scenario-based simulation have attracted increasing attention. Previous studies have demonstrated that manikin-based CPR training can significantly improve learners’ procedural skills and clinical performance, while scenario-based simulation training contributes to enhanced emergency response capability and overall performance in simulated clinical settings (5). The integration of scenario simulation and manikin practice provides learners with hands-on experiences that closely resemble real emergency environments, enabling them to engage in a continuous learning cycle of observation, reflection, and repeated practice within simulated contexts. This process not only deepens their understanding of emergency care knowledge but also improves skill proficiency and retention. However, existing studies have largely focused on medical or nursing students (9), with relatively limited research involving middle school students. Moreover, most previous studies have employed a single intervention approach, lacking systematic investigations that integrate manikin-based training with scenario-based drama review. In addition, the majority of current studies are based on non-randomized controlled designs, with insufficient analysis of the main effects and interaction effects of different teaching components, as well as a lack of follow-up assessments examining long-term skill retention. Therefore, there is a clear need to conduct rigorously designed randomized controlled trials among middle school students to systematically evaluate the effectiveness of a CPR training model that combines manikin-based practice with scenario-based drama review.

Accordingly, the present study aims to investigate the effectiveness of a CPR training approach that combines manikin-based practice with scenario-based drama review among middle school students, in comparison with conventional training methods. Through a randomized controlled trial design, this study will systematically evaluate differences in CPR knowledge acquisition, practical skill performance, and emergency response capacity across different training modalities. The findings are expected to provide a more effective intervention strategy for CPR education among middle school students and offer theoretical evidence as well as empirical support for first aid education practice and related policy development.

### 1.2 Study aims

The specific objectives of this study are to: (1) compare the effectiveness of a CPR training model that combines manikin-based practice with scenario-based drama review versus conventional training methods among middle school students; (2) evaluate the impact of this intervention on CPR-related outcomes, including knowledge acquisition, skill performance, self-efficacy (behavioral performance and self-evaluation), intention to perform bystander cardiopulmonary resuscitation, and course satisfaction; and (3) assess the long-term retention of CPR skills following the implementation of the intervention.

## 2 Methods and analysis

### 2.1 Study design and settings

This study is designed as a single-center, single-blind, 2 × 2 factorial cluster randomized controlled trial involving middle school students aged 11–18 years. The trial will be conducted in a middle school in Hohhot, Inner Mongolia Autonomous Region. This study setting offers favorable implementation conditions and organizational advantages: cluster allocation based on existing classes aligns with routine educational management, facilitates standardized delivery of training and follow-up assessments, and effectively reduces contamination associated with individual randomization, thereby enhancing both feasibility and internal validity. The factorial design allows for the independent evaluation of the main effects of manikin-based training and scenario-based drama review, as well as their interaction effect. This study includes Baseline assessment (T0), immediate post-first training assessment (T1), 3-month post-first training assessment (T2), immediate post-second training assessment (T3), 3-month post-second training follow-up (T4), to evaluate the short-term and long-term effects of the intervention.

This study was approved by Inner Mongolia Maternal and Child Health Care Hospital Approval Form of the Medical Ethics Committee [File No (2025) Lunshen K No. (021-1)].

### 2.2 Eligibility criteria

The inclusion criteria are as follows: (1) middle school students aged 11–18 years; (2) voluntary participation with informed consent obtained from both the participants and their legal guardians.

The exclusion criteria are as follows: (1) individuals with physical disabilities that limit motor activity; (2) individuals unwilling to cooperate or those who withdraw from the study during the study period.

### 2.3 Recruitment and procedures

Recruitment will commence in March 2026. The study population will consist of middle school students. Prior to study implementation, permission will be obtained from school authorities. The study objectives and procedures will be introduced to students through classroom-based presentations, and informed consent forms will be distributed to both students and their legal guardians. Participants will be formally enrolled after obtaining written informed consent from their guardians and voluntary assent from the students. Following enrollment, all participants will complete a baseline assessment and will then be allocated by class to different groups through randomization, after which they will receive the corresponding interventions.

### 2.4 Randomization and group allocation

This study adopts a cluster randomized design, with classes serving as the unit of randomization. The randomization process will be conducted in two stages, as follows:

#### 2.4.1 Stage 1: selection of eligible classes

First, among a total of 16 classes that meet the inclusion criteria within the school, 4 target classes will be randomly selected using Microsoft Excel. The procedure is as follows: a new worksheet will be created, with Column A assigned numbers from 1 to 16 as unique identifiers for each class. In Column B, the formula = RAND() will be used to generate random numbers for each class. These random numbers will then be copied and pasted as values to prevent subsequent changes. All classes will then be sorted in ascending order based on the values in Column B, and the first 8 classes will be selected as the study sample.

#### 2.4.2 Stage 2: random allocation to intervention groups

The selected 4 classes will then be randomly assigned to intervention groups using Excel. Specifically, in a new worksheet, Column A will be assigned numbers from 1 to 4 as unique identifiers for the included classes. In Column B, the = RAND() function will again be used to generate random numbers, which will subsequently be copied and pasted as values. The classes will then be sorted in ascending order based on Column B. According to the sorted order, classes will be sequentially allocated into four intervention groups: the 1st classes will be assigned to Intervention Group 1, the 2nd classes to Group 2, the 3rd classes to Group 3, and the 4th classes to Group 4. The class identifiers will remain in Column A, while the allocation will be determined by the ranking in Column B.

To ensure the validity of randomization, a single-blind design will be employed, whereby participants will be unaware of their group assignments, while only the intervention implementers will have access to the allocation information. In the event of a serious adverse event or when knowledge of group allocation is required to ensure participant safety, unblinding will be requested by the principal investigator and implemented after approval by the Ethics Committee, with the entire process fully documented. To maintain allocation concealment, the sealed envelope method will be used. Specifically, four opaque envelopes will be prepared, each labeled with a unique identifier corresponding to the class identifiers in Column A. Inside each envelope, a slip indicating the assigned group will be placed. The allocation information will remain sealed and concealed until the commencement of the intervention.

### 2.5 Intervention

All participants will receive standardized conventional cardiopulmonary resuscitation (CPR) training. Based on group allocation, additional intervention components will be implemented accordingly. Training will be conducted at the class level. The training program consists of the following three components: (1) conventional CPR training: a combination of video demonstration and instructor-led explanation will be used to systematically teach the fundamental principles and procedural steps of CPR, including chest compressions, rescue breathing, and key technical considerations (the training session lasts for 60 min). (2) Manikin-based training: under instructor supervision, students will perform CPR on manikin models, focusing on essential skills such as compression depth, compression rate, hand positioning, and ventilation technique. Real-time feedback will be provided by instructors to improve procedural accuracy and standardization (the training session lasts for 60 min). (3) Scenario-based simulation (drama-based review): typical emergency scenarios (e.g., sudden cardiac arrest occurring at school, at home, or in a public park) will be constructed. Students will participate in role-playing activities (e.g., rescuer, bystander, caller for help), performing the full CPR procedure within simulated contexts while also developing emergency response, communication, and psychological coping skills. To ensure the standardization and reproducibility of the scenario-based simulation training, standardized drama scripts were developed by the research team and are provided in Supplementary material 1. The scripts were designed around a complete first-aid response sequence, including identifying an emergency, assessing the environment and calling for help, evaluating the victim’s condition, calling the emergency medical service number 120, performing standardized CPR compressions and rescue breathing, and assessing resuscitation outcomes. This structure formed a complete prehospital emergency response loop and closely integrated real-life scenarios with key professional first-aid knowledge points, thereby enhancing students’ situational engagement and practical response capacity (the training session lasts for 60 min).

All instructors were certified Basic Life Support trainers by the American Heart Association (AHA) and received standardized pre-training in CPR operational skills and teaching procedures to ensure consistency in training content, instructional materials, and performance standards. A total of eight instructors were responsible for 160 participants, corresponding to an instructor-to-participant ratio of approximately 1:20. Four CPR manikins were used for classroom demonstration and hands-on practice, and an additional two manikins were designated for skills assessment (each participant was allocated approximately 15 min per CPR skills assessment session). In total, six CPR manikins were used throughout the study.

### 2.6 Study arms

All participants will receive standardized conventional CPR training. Based on random allocation, participants will then receive different forms of enhanced training or review interventions. Middle school students will be randomly assigned to one of the following four groups:

Group A (Manikin-based training + scenario-based drama review):

Phase 1: following conventional CPR video demonstration and theoretical instruction, students will undergo manikin-based CPR skills training, practicing key steps such as compression depth, rate, and rescue breathing under instructor guidance.

Phase 2: scenario-based drama review will be conducted by simulating emergency situations (e.g., sudden collapse on campus). Students will engage in role-playing (rescuer, bystander, caller for help) and complete the CPR procedure within these scenarios to reinforce skill application and emergency response capacity.

Group B (Manikin-based training only):

Phase 1: following conventional CPR video demonstration and theoretical instruction, students will receive manikin-based CPR skills training, focusing on repeated practice of compression depth, rate, and ventilation under instructor guidance. Phase 2: conventional CPR training (theoretical explanation and demonstration) will be provided.

Group C (scenario-based drama review only):

Phase 1: students will complete conventional CPR video demonstration and theoretical instruction without manikin-based practice. Phase 2: scenario-based drama review will be conducted through simulated emergency situations (e.g., sudden collapse on campus), with students participating in role-playing and completing CPR procedures to enhance practical application and emergency response skills.

Group D (control group):

Phase 1: students will complete conventional CPR video demonstration and theoretical instruction. Phase 2: conventional CPR theoretical instruction and standard procedural demonstration will be provided.

**Figure 1:**
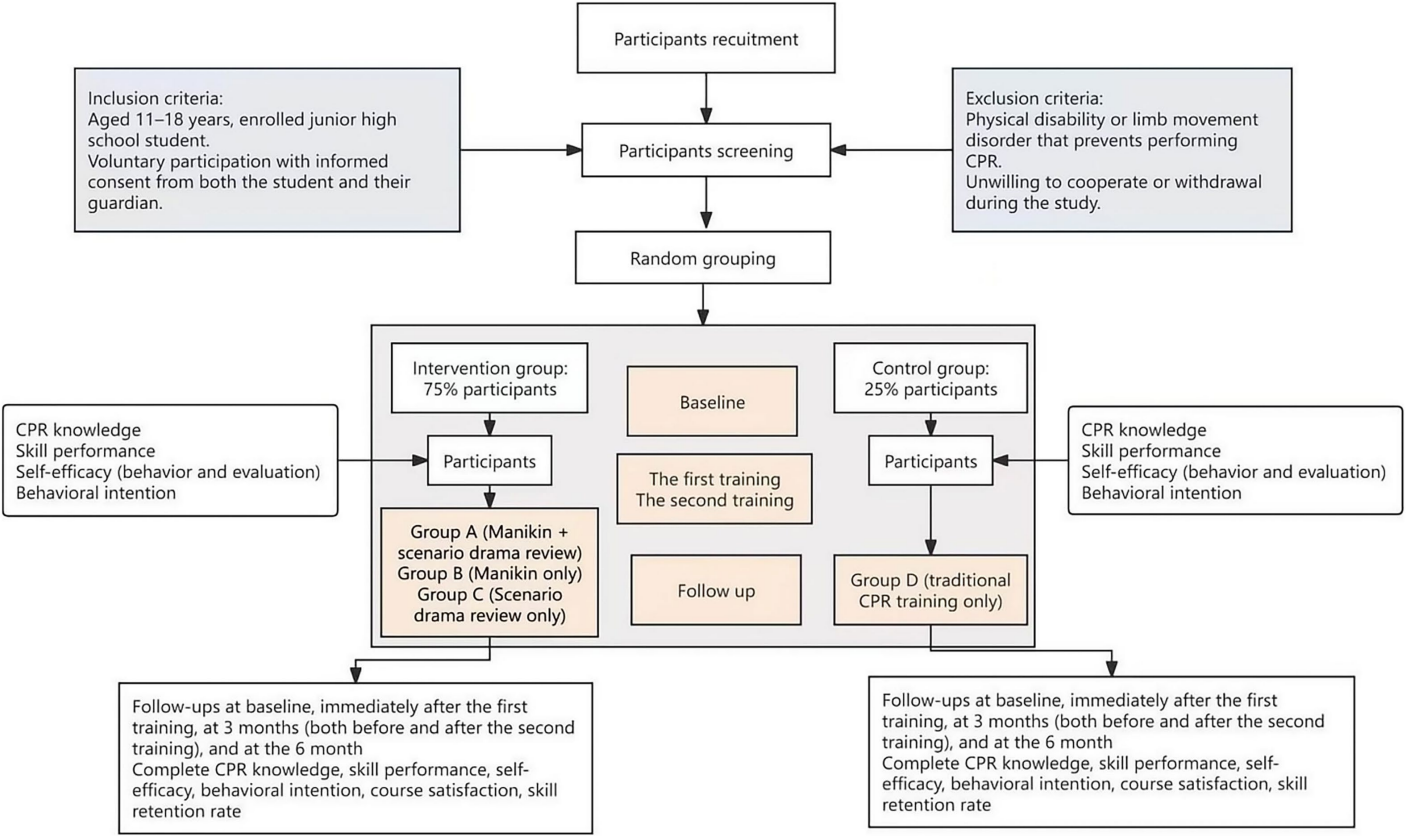
Flow chart of participants recruitment and study implementation.

To ensure consistency across groups, all training sessions will be delivered by uniformly trained instructors. The duration and overall instructional framework will be standardized, with differences limited to the inclusion or exclusion of manikin-based training and scenario-based drama review. The detailed study flowchart is presented in Figure 1.

### 2.7 Control condition

During the intervention period, the control group will receive only conventional CPR training, including theoretical instruction and procedural demonstration, without additional manikin-based practice or scenario-based drama review. The training duration will be kept consistent with that of the intervention groups to control for the potential influence of time on study outcomes.

### 2.8 Outcome measurements

#### 2.8.1 CPR knowledge assessment

This section consists of nine multiple-choice questions covering fundamental CPR knowledge, airway opening and rescue breathing, and assessment of resuscitation effectiveness (10). Each correctly answered question is scored as 1 point, while unanswered or incorrect responses receive 0 points. The total score is the sum of all items, ranging from 0 to 9, with higher scores indicating better knowledge.

#### 2.8.2 CPR skills assessment

CPR skills will be evaluated based on the assessment criteria developed by Zhao et al. (11). The evaluation includes eight components: environmental assessment, assessment of consciousness and activation of emergency medical services (EMS), recognition of cardiac arrest, patient positioning, chest compressions, airway clearance and opening, rescue ventilation, and the compression-to-ventilation ratio.

#### 2.8.3 Self-efficacy (recognition and evaluation)

The Basic Life Support Self-Efficacy Scale, originally developed by ESC 365 (12) and culturally adapted into Chinese by Zhang et al. (13), will be used. The scale comprises three dimensions [recognition and evaluation, CPR performance, and safe use of an automated external defibrillator (AED)] with a total of 18 items. In this study, only the “recognition and evaluation” dimension will be used. Each item is rated on a 4-point Likert scale ranging from “strongly disagree” (1 point) to “strongly agree” (4 points). The Cronbach’s *α* coefficient for each dimension of the scale exceeds 0.80, indicating good internal consistency.

#### 2.8.4 Behavioral intention

Behavioral intention to perform bystander CPR will be assessed using a scale developed by Zheng et al. (14). The scale includes four dimensions, of which only the behavioral intention dimension will be used in this study.

Items are rated on a 5-point Likert scale: “strongly disagree” (1 point), “disagree” (2 points), “uncertain” (3 points), “agree” (4 points), and “strongly agree” (5 points). The scale demonstrates excellent reliability, with Cronbach’s *α* coefficients exceeding 0.90 and split-half reliability greater than 0.80 across dimensions, indicating high internal consistency.

#### 2.8.5 Course satisfaction (CSQ-3)

Participant satisfaction with the intervention will be assessed using the Client Satisfaction Questionnaire-3 (CSQ-3) (15, 16). This scale consists of three items designed to evaluate satisfaction with the received services, focusing on perceived quality, usefulness, and willingness to recommend the program to others.

The CSQ-3 has demonstrated good reliability and validity across different populations, with reported Cronbach’s *α* values typically exceeding 0.85. Each item is rated on a 4-point Likert scale, where 1 = strongly disagree and 4 = strongly agree. Higher scores indicate greater satisfaction with the program.

#### 2.8.6 Skill retention rate

CPR skill retention will be assessed at 3 months post-training. The retention rate will be calculated as: retention rate (%) = (follow-up score/immediate post-training score) × 100%. This measure will be used to evaluate the long-term retention of CPR skills among middle school students.

### 2.9 Blinding and assessment quality control

#### 2.9.1 Blinding of participants and intervention providers

This study employed a cluster randomized controlled design. Participants were not informed of the specific differences between the study groups, such as whether they received simulation-based training or scenario-based role-playing training. However, intervention providers were aware of the intervention content and therefore could not be blinded. This limitation is inherent to the study design.

#### 2.9.2 Blinding of outcome assessors

All outcome assessments were independently conducted by two emergency medicine instructors who had received standardized training. These assessors were not involved in group allocation, intervention implementation, or data management. In addition, they had no access to participants’ group assignments or previous assessment results during the evaluation process. Practical skills were evaluated using standardized scoring criteria, while questionnaire data were collected anonymously. Data entry, management, and statistical analyses were performed by designated research personnel who remained blinded to group allocation throughout the study.

#### 2.9.3 Assurance of inter-rater reliability

Before the formal assessment, the two assessors underwent standardized training to ensure consistency in scoring. Ten CPR performance videos from individuals outside the study sample were used for pilot assessment. The intraclass correlation coefficient was calculated to evaluate inter-rater agreement, and an intraclass correlation coefficient of at least 0.85 was required before formal assessment. During the formal assessment, 5 % of the operation videos were randomly selected for repeat evaluation to monitor inter-rater reliability. If the intraclass correlation coefficient fell below 0.80, additional training and recalibration of the scoring criteria were conducted.

### 2.10 Sample size calculation

This study is a two-factor factorial cluster randomized controlled trial, with “CPR knowledge and skills scores” as the primary outcome. Based on the literature, a significance level of 5% (*α* = 0.05) and a statistical power of 80% (*β* = 0.20) were adopted. Sample size estimation was informed by data from previous studies. It was assumed that the mean total CPR score (combined knowledge and skills score rate) would be 80% in the intervention group and 65% in the control group, with a standard deviation of 15% in both groups, corresponding to an effect size of *d* = 1.0. Assuming a 15% difference in the primary outcome (main effect), G*Power 3.1 was used for sample size calculation, indicating that a total of 128 participants would be sufficient for multivariate regression analysis (effect size = 0.25, power = 0.80, critical *F* = 3.921). Considering a potential attrition rate of 20%, the final minimum sample size was set at 160 participants. This study also takes into account the design effect resulting from the correlation among subjects within clusters. The design effect is calculated using the formula DE = 1 + (m − 1)*ρ*, where m represents the average number of subjects per cluster, and ρ represents the intracluster correlation coefficient (ICC) (17). In cluster randomized trials, the design effect is commonly used to adjust the sample size required for an individual randomized trial to the sample size required for a cluster-randomized design. The final minimum sample size for this study was set at 160 participants, which represents a 25% overall inflation factor added to the 128 participants required at the individual level. This inflation factor accounts for both the cluster-randomized design effect and the potential impact of dropouts or invalid data. Based on the average cluster size in this study and the ICC estimated from previous similar studies, the design effect did not exceed 1.25 (18). Therefore, after adjusting for the design effect, a sample size of 160 participants (selecting 4 classes, with 40 participants per class) would still provide no fewer than 128 valid cases, thereby meeting the requirements for testing the primary effect.

### 2.11 Data collection and management

Data will be collected at the following time points: (1) baseline assessment (T0): conducted prior to the first training, including CPR knowledge score, skills performance score, self-efficacy (recognition and evaluation), and behavioral intention; (2) immediate post-first training assessment (T1): conducted immediately after the first training session, including CPR knowledge score, skills performance score, self-efficacy (recognition and evaluation), behavioral intention, and course satisfaction; (3) 3-month post-first training assessment (T2), conducted prior to the second training session, including CPR knowledge score, skills performance score, self-efficacy (recognition and evaluation), and behavioral intention; (4) immediate post-second training assessment (T3): conducted immediately after the second training session, including CPR knowledge score, skills performance score, self-efficacy (recognition and evaluation), behavioral intention, and course satisfaction; (5) 3-month post-second training follow-up (T4), only questionnaires will be collected, including CPR knowledge score, skills performance score, self-efficacy (recognition and evaluation), behavioral intention, course satisfaction, and skill retention rate. The skills performance scores will be assessed by instructors who rotate across the four classes, such that each instructor trains and evaluates the skills for all classes in turn. This rotation minimizes rater bias and ensures consistency and fairness in skill assessment.

All data will be collected and managed using an electronic data capture platform (e.g., Wenjuanxing). Each participant will be assigned a unique identification code (ID), and all data will be anonymized. Data will be stored in encrypted formats, with access restricted to the research team to ensure participant privacy and data security. Logical checks and outlier alerts will be implemented during data entry to enhance data quality.

### 2.12 Data monitoring and quality assurance

As this study primarily involves CPR skills training, appropriate safety measures will be implemented during each training session to ensure that students are not injured during simulated practice. All participants will perform CPR under the supervision of experienced instructors to ensure safety. Trial monitoring will be conducted by a supervisory team from the coordinating center, which operates independently of the on-site investigators and reports to the steering committee. The data management system will automatically validate input data. If any recorded values fall outside acceptable ranges, warning messages will be generated, prompting users to verify and correct the entries.

### 2.13 Data analysis plan

This study adopted a 2 × 2 factorial cluster randomized controlled trial design, with class as the unit of random allocation and individual students as the unit of analysis. All statistical analyses will account for clustering at the class level and the correlation among repeated measurements from the same participant. Three analysis sets will be predefined: the full analysis set, the per-protocol set, and the safety analysis set. The full analysis set will serve as the primary analysis population and will include all randomized participants, who will be analyzed according to their originally assigned groups, following the intention-to-treat principle. Participants will not be excluded from the primary analysis because of intervention non-completion, absence from training sessions, or withdrawal during the intervention period, nor will their originally assigned group be changed. The per-protocol set will be used for robustness analyses and will include only participants who complete the predefined core intervention components and complete the primary outcome assessment. The safety analysis set will include participants who receive at least one intervention session or participate in at least one training activity, and will be used for descriptive analyses of adverse events and safety-related information. Two binary intervention factors are specified: Factor 1, initial training method, including lecture-only training versus lecture plus manikin practice; and Factor 2, booster reinforcement method, including traditional review versus scenario-based simulation. The four factorial groups correspond to Classes 13, 1, 10, and 12, respectively.

The research team will strictly record and distinguish participants who withdraw during the intervention period, defined as participants who stop receiving the assigned training content after randomization; those who do not complete the intervention, defined as participants who fail to meet the predefined core course completion criteria; those who are absent from one or more scheduled training sessions; and those lost to follow-up, defined as participants who do not complete the outcome assessments at the prespecified time points. For participants who do not complete the intervention or are absent from training sessions, the research team will still make every effort to collect subsequent outcome data, unless the participant or their guardian explicitly withdraws consent for continued participation or further data collection. The primary analysis will continue to follow the intention-to-treat principle, whereby all randomized participants will be included in the analysis according to their originally assigned groups. Per-protocol analysis will be conducted as a sensitivity analysis to assess the influence of intervention adherence on the study findings.

Baseline characteristics will be summarized by study group. Continuous variables will be presented as means and standard deviations or medians and interquartile ranges, depending on their distribution. Categorical variables will be presented as frequencies and percentages. Baseline variables with important clinical or educational relevance, as well as variables showing apparent imbalance across study groups, will be adjusted for as covariates in subsequent models.

Continuous outcomes will be analyzed using linear mixed-effects models. Binary outcomes will be analyzed using generalized linear mixed-effects models, and ordinal outcomes will be analyzed using ordinal logistic mixed-effects models. The models will include both fixed and random effects. Fixed effects will include the two intervention factors, time, the interaction between the two intervention factors, the interactions between each intervention factor and time, and the three-way interaction among the two intervention factors and time. When necessary, baseline outcome values and other prespecified covariates will also be included. Random effects will include a class-level random intercept to account for the correlation among students within the same class, as well as an individual-level random intercept to account for the correlation among repeated measurements from the same participant over time.

For repeated-measures data, within-participant correlation will be addressed through the inclusion of an individual-level random intercept. If the number of measurement time points and sample size allow, an unstructured covariance structure will be prioritized. If the model fails to converge or parameter estimates are unstable, compound symmetry or first-order autoregressive covariance structures will be considered as alternatives. The final model will be selected based on model fit indices and study design considerations. Model selection will consider the Akaike information criterion, Bayesian information criterion, model convergence, and the interpretability of parameter estimates.

Factorial effects will be analyzed according to the principle of interaction-first interpretation. For the primary outcome, the interaction between the two intervention factors and the three-way interaction among the two intervention factors and time within the repeated-measures framework will first be tested. If the interaction is statistically significant or shows a practically meaningful trend, simple-effects analyses will be conducted to estimate the effects of different intervention combinations at each time point. If no clear interaction is observed, the main effects of the two intervention factors will be interpreted. For intervention effects that vary over time, the interaction terms between intervention factors and time will be treated as key parameters for evaluating changes in intervention effects.

Adjusted effect estimates and their 95% confidence intervals will be reported. For continuous outcomes, between-group mean differences or standardized effect sizes will be reported. For binary outcomes, odds ratios or relative risks with 95% confidence intervals will be reported. For ordinal outcomes, corresponding ordinal odds ratios will be reported. The intraclass correlation coefficient and design effect will be calculated to quantify clustering effects. The robustness of the results will be examined through per-protocol analysis and multiple imputation.

For missing data, the number, proportion, and reasons for missingness will first be reported by study group. If outcome data are missing, multiple imputation will be performed under the missing-at-random assumption. Each imputed dataset will be analyzed separately, and the estimates will then be pooled. To assess the potential impact of post-randomization differential attrition on the study conclusions, sensitivity analyses will be conducted. If there are apparent differences across study groups in intervention-period withdrawal rates, absence rates, intervention non-completion rates, or loss-to-follow-up rates, their potential contribution to selection bias and their possible influence on estimates of intervention effects will be discussed in the interpretation of the results.

Given that this is a pilot study, the sample size was primarily determined based on implementation feasibility. A lack of statistical significance in interaction tests will not necessarily rule out the presence of practically meaningful interaction effects. Therefore, the findings will be interpreted comprehensively by considering effect sizes, confidence intervals, intervention adherence, and implementation feasibility. All statistical tests will be two-sided, and a *p* value of < 0.05 will be considered statistically significant. Statistical analyses will be conducted using SPSS 27.0 and R 4.5.2 (packages lme4, emmeans, and mice).

## 3 Discussion

OHCA is a major global public health issue, and its high mortality rate is closely associated with insufficient public first aid capacity. As potential “first witnesses,” middle school students’ CPR skills are directly related to the quality of initial emergency response in sudden events. However, conventional CPR training primarily relies on lectures and demonstrations, lacking contextualization and hands-on practice, which limits its ability to meet the demands of real-life emergency situations in terms of skill proficiency and rapid response capability. The integration of manikin-based training with scenario-based simulation provides a promising approach to overcoming the limitations of traditional teaching methods.

In this study, a CPR training model combining manikin-based practice with scenario-based drama review is implemented to enhance knowledge internalization and skill transfer through a dual approach of “hands-on practice + scenario simulation.” Compared with single-method training, this model not only focuses on procedural skills but also emphasizes decision-making and psychological adaptability in complex situations, thereby improving learners’ comprehensive response capabilities in real emergency settings.

In terms of study design, this research adopts a single-blind cluster randomized controlled trial with a 2 × 2 factorial design, allowing for the simultaneous evaluation of the main effects and interaction effects of manikin-based training and scenario-based drama review. This design enables a more systematic analysis of the effectiveness and synergistic effects of different instructional strategies. A total of 160 middle school students will be included and randomly assigned to four groups in a 1:1:1:1 ratio, each receiving different combinations of interventions. Assessments will be conducted at Baseline assessment (T0), immediate post-first training assessment (T1), 3-month post-first training assessment (T2), immediate post-second training assessment (T3), 3-month post-second training follow-up (T4). Outcome measures will include CPR-related knowledge, skill performance, behavioral intention, and skill retention rate, providing a comprehensive evaluation of both immediate and sustained intervention effects.

The innovations of this study are reflected in the following aspects. First, it adopts a combined intervention strategy integrating manikin-based training with scenario-based drama review, strengthening the integration of skill acquisition and contextual application. Second, the use of a factorial design optimizes the evaluation of intervention strategies, improves research efficiency, and enables the identification of the mechanisms and interaction effects of different intervention components.

Several limitations should be acknowledged. First, the study is conducted in a single center within one region, which may limit the generalizability of the findings. Second, some outcome measures rely on self-reported scales, which may introduce subjective bias. Third, although 3-month follow-up is included, the study primarily reflects short- to medium-term effects; longer-term skill retention and behavioral translation require further investigation.
