# Supplementary material for: Manikin-based practice combined with scenario-based simulation for cardiopulmonary resuscitation training among middle school students: study protocol for a cluster randomized controlled trial
Source: Front Public Health. 2026 Jul 8;14:1878058. doi: 10.3389/fpubh.2026.1878058 (PMC13388850; doi:10.3389/fpubh.2026.1878058)
Supplement: Supplementary file 1 [file Table_1.DOC]

**Supplementary material 1**

The script of a sitcom is divided into three types：Script for a Family,Script for a Park-Setting CPR Scenario Play,Script for a School-Based CPR Role-Play.

**Script for a Family CPR Role-Play (6 Participants)**

Scene: Grandpa suffers a sudden cardiac arrest while getting up in the early morning

Characters: Dad (Lead Rescuer), Mom (Chest Compression Assistant), Daughter Xiaohong (Calls 911), Son Xiaoming and Neighbor 1 (Mouth-to-Mouth Resuscitation Assistants), Grandpa (Patient)

Act 1: Grandpa Suffers a Sudden Heart Attack—Can He Be Saved?

Scene: One early morning, as Grandpa is about to get out of bed, he feels unwell. He experiences severe pain in his left chest, as if it were on fire.

Grandpa (in pain): Ouch, ouch! (He clutches his left chest and kneels down in distress)

Father (seeing Grandpa’s distress from a distance and rushing over): Dad! Dad! What’s wrong with you!

Grandpa: Help me... (closes his eyes and lies on the ground)

Act 2: Preparing for First Aid

Father (looking around and waving his hands): Help! Help! Someone, please come quickly!

(Others rush onto the scene): What’s going on? What happened?

Father (anxiously): My father is having a heart attack. He needs immediate first aid—I need your help!

(Others): Of course!

Father directs Neighbor 1: Please help me move these items out of the way to prevent secondary injuries from bumping into them during the rescue.

Neighbor 1 (responding quickly): Okay! (Makes a motion to move the items and steps aside)

Father (standing, scanning the area): The scene is clear and safe! (Then kneels on the right side of his father, gently pats his grandfather’s shoulders, and calls out close to his ear): Dad! Dad! Can you hear me?

Mother (kneeling on Grandpa’s left side, pressing lightly with her fingertips together on one side of his Adam’s apple to check for a carotid pulse, counting as she checks): 1001, 1002, 1003, 1004, 1005, 1006, 1007—Dad has no breathing and no carotid pulse! His chest isn’t rising and falling!

Mother: Hurry! Xiaohong, go call 911.

Xiaohong (calling 911): Hello! This is Building 1, Unit 3, Sunshine Community. My grandfather suddenly collapsed; he’s unconscious and not breathing. He has a history of heart disease. We need medical assistance!

Scene 3: Resuscitation Begins

(Neighbors and parents position Grandpa’s body so his head, neck, and torso are in a straight line, with his arms at his sides)

Dad (kneeling to Grandpa’s right, knees shoulder-width apart, unbuttoning Grandpa’s outer garment to fully expose the chest): Son, do you remember how to perform chest compressions?

Xiaoming: Place the heel of one hand on the patient’s skin, stack the other hand on top of the first, clasp your hands tightly, keep your elbows straight, and press down vertically with both arms, depressing the sternum 5–6 centimeters. After each compression, allow the chest to fully recoil, but do not remove your hands from the chest wall.

Dad: Excellent, thank you, Xiao Ming. (Begins compression using the correct method)

Mom: The compression rate should be 100–120 times per minute. Perform 30 compressions per cycle, followed by two rescue breaths after every 30 compression. Honey, let me take over after you’ve done two cycles.

Dad (while performing compression): Okay.

Dad (after 30 compression): It’s time for rescue breaths (about to proceed directly to rescue breaths)

Xiaohong: Dad! You can’t just go straight to rescue breaths! You need to check Grandpa’s neck and mouth for injuries, see if he has any removable dentures, tilt his head to one side to clear secretions from his nose and mouth, and open his airway before you can perform rescue breaths!

Dad: Oh, right, right, right! I was so anxious I almost forgot (follows Xiao Hong’s instructions to check and clear the nose and mouth of foreign objects; after finishing, places one hand on Grandpa’s forehead and tilts his head back, while placing the other hand under his jaw and lifting it up. Once in position, pinches Grandpa’s nose with his thumb and index finger, blows air into his mouth, releases the nose pinch, and repeats the process)

(After Dad completes two rounds, Mom takes over for two more rounds. If time permits, the two neighbors should also perform two rounds.)

Scene 4: Grandpa slowly regains consciousness; the rescue is a complete success.

Mom (after completing the second round of CPR, checking for a carotid pulse and observing the chest): Grandpa has regained a carotid pulse and is breathing on his own!

Neighbor 1 (crouching down to check on Grandpa, touching his hand): The resuscitation worked! Look—Grandpa’s complexion, lips, and skin are turning pink, and his hands are getting warm.

Xiao Ming (helping Grandpa adjust his clothes): Grandpa! You’re awake. Don’t worry—there was just a little scare earlier, but there’s nothing to be concerned about. We’ll all be right here with you!

**Script for a Park-Setting CPR Scenario Play (6 Characters)**

Scene: An elderly man suddenly goes into cardiac arrest while taking a morning walk

Characters: Xiao Ze (Lead Rescuer), Xiao Ya (Chest Compression Assistant), Xiao Xuan (Calls 911), Xiao Lin (Rescue Breathing Assistant), Xiao Hao (Evacuates Bystanders and Maintains Order), Elderly Man (Patient)

Scene:

Act 1: Playing in the Park, Elderly Man Suffers Sudden Collapse

Time: Weekend Afternoon

Setting: By the park lawn, several middle school students are playing together; an elderly man is taking a walk in the park

(Curtain rises. Xiao Ze, Xiao Ya, Xiao Xuan, and two other students are happily walking and chatting in the park. An elderly man is walking slowly on the path nearby.)

Elderly Man: (Suddenly stops, clutches his chest with both hands, furrows his brow, and turns pale) Oh my... my chest feels so tight... it hurts so much I can’t breathe...

(After speaking, the elderly man collapses to the ground, closes his eyes, and lies motionless)

Xiao Ze: (The first to notice, immediately shouts) Help! Someone’s collapsed! Everyone, come quickly!

(The students immediately ran over and gathered around the elderly man, their faces filled with concern.)

Scene 2: Staying Calm, Dividing Tasks, and Preparing for First Aid

Xiao Ze: (Crouching beside the elderly man, shouting loudly) Grandpa! Grandpa! Can you hear me? (He gently tapped the old man’s shoulder, but the old man showed no reaction.) (Kneeling to the old man’s left, he pressed his fingertips together and gently pressed against the side of his Adam’s apple to check for a carotid pulse, counting as he checked): 1001, 1002, 1003, 1004, 1005, 1006, 1007—the old man has no breathing and no carotid pulse! No chest movement!

Xiao Ze: (Standing up calmly to give instructions) Everyone, stay calm! We’ve learned CPR—let’s divide up the tasks and help out!

1. Xiao Xuan: Call 911 immediately and give the exact address: next to the central lawn in City Park. An elderly man has collapsed—he’s not breathing and is unconscious. Tell them to hurry!

2. Xiao Hao: You go clear the area of bystanders. Don’t let people crowd around—keep the airway clear!

3. Xiao Lin: Get ready to help me clear the old man’s mouth!

Everyone: Got it!

(Xiao Xuan immediately takes out his phone to call 120. Xiao Hao runs quickly to the park office and politely clears the onlookers. Xiao Lin stands by, ready.)

Xiao Ze: (Speaking loudly) The scene is safe! We’re starting CPR immediately!

Scene 3: Standard Procedure—Performing CPR

(Xiao Ze and Xiao Ya work together to lay the elderly man flat on the ground, aligning his body so his head, neck, and torso are in a straight line. They place his arms at his sides and loosen his collar and belt.)

Xiao Ze: (Kneeling beside the elderly man, he interlaces his fingers, clenches his hands, and places the heels of his palms in the center of the man’s chest.) He contacts the patient’s skin with the heels of his palms, keeps his elbows straight, and presses down vertically with his arms, depressing the sternum by 5–6 centimeters. After each compression, he allows the chest to fully recoil, but keeps his hands in contact with the chest wall.

(Xiao Ze begins compressing at a steady pace, quietly counting aloud: 1, 2, 3… 30)

Xiao Ya: We’ve completed 30 compressions. Let’s prepare for rescue breathing!

Xiao Lin: (Hurrying forward) I’ll check Grandpa’s neck and mouth for injuries and removable dentures. I’ll also tilt his head to one side, clear secretions from his nose and mouth, and open his airway before we can perform rescue breathing!

(Xiaolin places one hand on his forehead and the other under his chin, tilting his head back to open the airway)

Xiaolin: (Pinching his nose, she gently blows two breaths into his mouth, watching his chest rise) Okay, rescue breathing complete!

(Xiaoze continues compressions; after two cycles, he’s getting a bit tired)

Xiaoya: I’ll take over for you! (She immediately squats down and continues the compressions using the same method)

Scene 4: Successful Resuscitation, the Elderly Man Regains Consciousness

Xiaoya: He has a pulse! (The elderly man slowly opens his eyes, gently wiggles his fingers, and his complexion gradually becomes rosy)

Elderly Man: (Weakly) I… what happened to me…

Xiao Ze: Grandpa, you fainted just now, so we performed CPR on you. Don’t worry—it was just a minor incident. We’re all here with you!

**Script for a School-Based CPR Role-Play (6 Participants)**

Scenario: Xiao Hua Suffers Sudden Cardiac Arrest After Running Drills

Roles: Homeroom Teacher (Lead Rescuer), Math Teacher (Chest Compression Assistant), Student A (Calls 911), Student B and Student C (Mouth-to-Mouth Resuscitation Assistants), Xiao Hua (Patient)

Act 1: Xiao Hua Collapses After Running Drills—The Cause Is Sudden Cardiac Arrest!

Scene: After the recess exercises, everyone returns to the classroom. The math teacher notices that Xiao Hua hasn’t come back yet.

Math Teacher: Has everyone returned? We’re about to start class.

Student A: Teacher, Xiao Hua isn’t back yet.

Math Teacher: What’s going on with him? Did he take a sick day?

Student B: No, he said he wasn’t feeling well before the running drills. I don’t know how he is now.

Xiao Hua (enters the classroom looking distressed): Ouch, ouch! (Covers his left chest with his hand and kneels down in pain)

Math Teacher (seeing Xiao Hua is unwell, rushes over): Xiao Hua! Xiao Hua! What’s wrong with you!

Xiao Hua: I have congenital heart disease. Help me... (Closes his eyes and lies on the floor)

Scene 2: Preparing for First Aid

Math Teacher (looking around): Quick, go get the homeroom teacher!

Student C: Okay, I’ll go right now! (runs out)

Math Teacher (anxiously): Everyone, Xiao Hua has suddenly suffered a heart attack and needs first aid. I need your help!

Remaining students: Yes, teacher!

Math Teacher directs Students A, B, and C: Please help me move the desks and chairs out of the way to prevent Xiao Hua from getting injured by them during the rescue and causing secondary harm.

Students A, B, and C (responding quickly): Okay! (They make motions to move the furniture and step back to the side)

Math Teacher (standing and scanning the area): Confirming the scene is safe! (Then kneels on Xiao Hua’s right side, gently pats his shoulders, and shouts into his ear): Xiaohua! Xiaohua! Can you hear me?

(Student C brings the homeroom teacher onto the scene)

Homeroom Teacher (kneeling on Xiaohua’s left side, pressing lightly with the tips of two fingers on one side of the Adam’s apple to check for carotid pulse, counting as she checks): 1001, 1002, 1003, 1004, 1005, 1006, 1007. Xiao Hua has no breathing and no carotid pulse! His chest isn’t rising and falling!

The rest of the students (fussing anxiously and whispering to each other): What should we do? What should we do? / Hurry up and save him! / Should we call 911?

Homeroom Teacher: Stay calm! Follow my instructions. (Pointing at Student A) You use my phone to call 120.

Student A (taking the homeroom teacher’s phone to call 120): Hello! This is Class 3, Grade 8 at Experimental Middle School. We’re on the third floor of the main academic building, east wing. My classmate suddenly collapsed; he’s unconscious and not breathing. He has congenital heart disease. We request medical assistance!

Scene 3: Resuscitation Begins

(The math teacher and homeroom teacher position Xiao Hua’s body so that his head, neck, and torso are in a straight line, with his arms at his sides.)

Homeroom Teacher: (Kneeling to Xiao Hua’s right, knees shoulder-width apart, unbuttoning his jacket to fully expose his chest, and explaining the steps while performing chest compressions): During resuscitation, use the heel of your palm to make contact with the patient’s skin. Place one hand on top of the other, with the back of one hand resting on the other. Keep your hands clasped tightly and your elbows straight. Press down vertically with both arms, causing the sternum to depress by 5–6 centimeters. However, since you are still young, a compression depth of 5 centimeters is sufficient. Allow the chest to fully recoil after each compression, but do not remove your hands from the chest wall.

Math Teacher: Right. The compression rate should be 100–120 compressions per minute. Perform 30 compressions per cycle, followed by two rescue breaths after every 30 compressions. Homeroom Teacher, after you’ve done two cycles, let me take over.

Homeroom Teacher (while performing compressions): Okay.

Class Teacher (after 30 compressions): It’s time for rescue breaths (about to proceed directly to rescue breaths)

Student B: Teacher! You can’t go straight to rescue breaths! Xiao Hua is wearing a retainer—it might dislodge and block his airway!

Class Teacher: Oh, right! I was so anxious I almost forgot (checks and clears foreign objects from Xiao Hua’s mouth and nose, removes the retainer; after finishing, places one hand on Xiao Hua’s forehead and tilts the head back, while placing the other hand under Xiao Hua’s jaw and lifting it up. Once in position, pinches Xiao Hua’s nose with thumb and index finger, blows air into the mouth, releases the nose, and repeats once more)

(After the homeroom teacher completes two rounds, the math teacher takes over and performs two more rounds.)

Scene 4: Xiao Hua slowly regains consciousness; the resuscitation is a success.

Math Teacher (after completing the second round of CPR, checks the carotid pulse again and observes the chest): Xiao Hua’s carotid pulse has returned, and she’s breathing on her own!

(The students cheer.)

Student A (crouching down to examine Xiao Hua, touching his hand): The resuscitation worked! Look, Xiao Hua’s complexion, lips, and skin are turning pink, and his hands are getting warm.

Student B (helping Grandpa organize his clothes): Xiao Hua! You’re awake. Don’t worry—there was just a little accident earlier, but there’s nothing to be concerned about. We’re all here for you!

Class Teacher: Thank you all for your help. You made the right call and came to me right away—you did a great job! In the future, if any of you feel unwell, please make sure to take a sick day. Your health is far more important than running drills!

# References
